# Corona discharge characteristics of cylindrical electrodes in a two-stage electrostatic precipitator

**DOI:** 10.1016/j.heliyon.2020.e03334

**Published:** 2020-02-19

**Authors:** Tsrong-Yi Wen, Jiann-Lin Su

**Affiliations:** a Department of Mechanical Engineering, National Taiwan University of Science and Technology, Taiwan; b High Speed 3D Printing Research Center, National Taiwan University of Science and Technology, Taiwan

**Keywords:** Electrical engineering, Electrical systems reliability, Plasma physics, Surface property, Electrical property, Air quality, Electrostatic precipitator, Cylinder electrode, Corona discharge, Oxidation

## Abstract

Electrostatic precipitator (ESP) is an electrohydrodynamic-based air filter that charges particles based on corona discharge and collects particles by induced electrostatic forces. Inducing corona discharge requires strong electric fields that, however, bring reliability issues because of oxidation. This paper presents the characteristics of an ESP that uses the cylindrical corona electrodes whose longitudinal axis is perpendicular to the surface of the ground electrode. The characteristics include the current-voltage curve, the surface oxidation of the cylindrical corona electrodes, and the element analysis. The characteristics are presented with respect to the pitch and diameter of the cylindrical corona electrodes. The results show that the characteristics mentioned above can correlate to the electric fields around the cylindrical corona electrodes. Stronger electric field around the cylindrical corona electrode results in higher collection efficiency, more oxidation on the cylindrical corona electrode, and shorter life of the cylindrical corona electrode.

## 1 Introduction

Aerosol particles have been confirmed to be severely adverse to public health [1] and have caused millions of death a year worldwide [2]. Filtering aerosol particles thus becomes an important issue from the epidemiology point of view. Electrostatic precipitator (ESP) is one of the devices that are used to filter aerosol particles.

**Figure 1:**
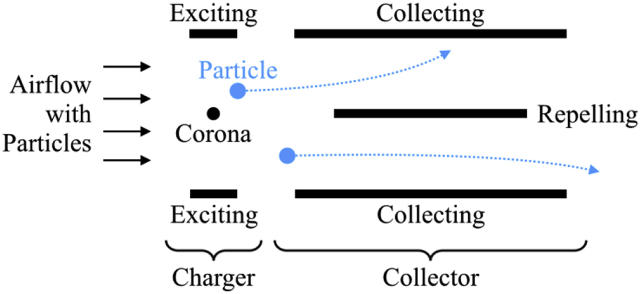
The schematic of a traditional two-stage ESP.

ESPs work based on electrohydrodynamics, including particle charging and particle transport [3]. Figure 1 shows the schematic of a traditional two-stage ESP that consists of a charger and a collector. The charger consists of one corona electrode (high-voltage) and two exciting electrodes (grounded), creating an extremely strong electric field around the corona electrode to make the corona discharge happen. The collector includes one repelling electrode (high-voltage) and two collecting electrodes (grounded), also creating a strong electric field in-between. Incoming particles get charged by the effects of corona discharge when passing by the charger. In the collector, instead of moving straightforward, the charged particles would move toward the collecting electrodes because of the induced electrostatic forces. Consequently, the charged particles are collected on the collecting electrodes.

Based on the Deutsch-Anderson equation, the collection efficiency of an ESP depends on several factors, such as corona voltage, airflow rate, and the properties of particles [4]. Of the properties of a particle, the number of charges a particle carries is particularly important and is dependent on the electric field around the corona electrode [5]. When the electric field around the corona electrode is stronger (or the corona current is higher), particles can get charged more efficiently, and thus the collection efficiency can be higher [6, 7, 8, 9].

The electric field around a corona electrode strongly depends on the curvature difference and the voltage difference between the corona electrode and the exciting electrode. A sharp corona electrode has a high tip curvature, and therefore, needle- or cylinder-plate charger can create a stronger electric field when compared with a wire-plate charger [10, 11]. El-Mohandes *et al.* showed that the corona current is higher at the same corona voltage when the number of the corona needles is fixed at six and the pitch (gap) between adjacent corona needles is larger [12]. Rong *et al.* also indicated that a large pitch of the corona electrodes can result in a strong electric field [13]. Although inducing a strong electric field helps to improve the collection efficiency, a strong electric field also speeds up the performance degradation of the corona electrodes because of the oxidation problems. Kim *et al.* explained how electrical discharge and oxidation are related to each other [14]. Selivonin *et al.* demonstrated that the performance degradation of the plate electrodes used in dielectric barrier discharge [15]. Nevertheless, the performance degradation of the cylindrical electrodes has not been presented.

This paper presents the characteristic changes of the cylindrical corona electrodes that are used in an ESP after long-term operation, including the current-time curves, the surface oxidation images (scanning electron microscope, SEM), and the element analysis. Excluding those known in the Deutsch-Anderson equation, this paper focuses on the characteristic changes with respect to the diameter and the pitch of the cylindrical corona electrodes.

## 2 Methods

### 2.1 Experimental setup

**Figure 2:**
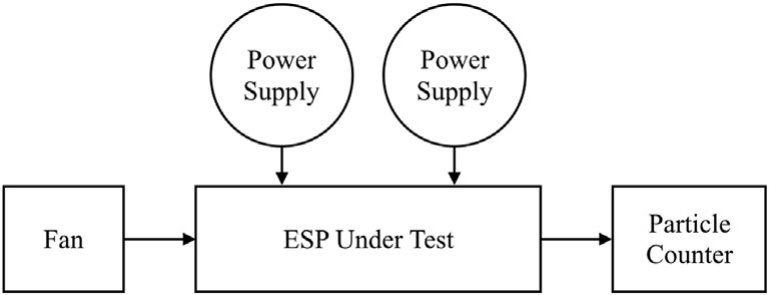
The schematic of the experimental setup.

Figure 2 shows the schematic of the experimental setup. Air and ambient particles are drawn into the ESP under test by a traditional rotary fan at a constant rate of 4.3×10^−3^ m^3^/s (258 LPM). Two DC power supplies (YSTC-HVPS) provide the positive high voltages to the ESP under test (charger and collector) independently. There is a particle counter (MSP-1000XP) placed downstream of the ESP under test to measure the numbers of particles in terms of particle size. Once the numbers of particles are measured, the collection efficiency η can be calculated by (1).

(1) $$\eta =\left(1-\frac{N_{\text{ESP on}}}{N_{\text{ESP off}}}\right)\times 100\%$$

where $N_{\text{ESP on}}$ and $N_{\text{ESP off}}$ are the numbers of particles when the ESP under test is turned on and turned off (background number of particles), respectively. Note that the collection efficiency shown in (1) is valid for specific particle size, i.e., the collection efficiency varies from one particle size to another.

### 2.2 ESP under test

**Figure 3:**
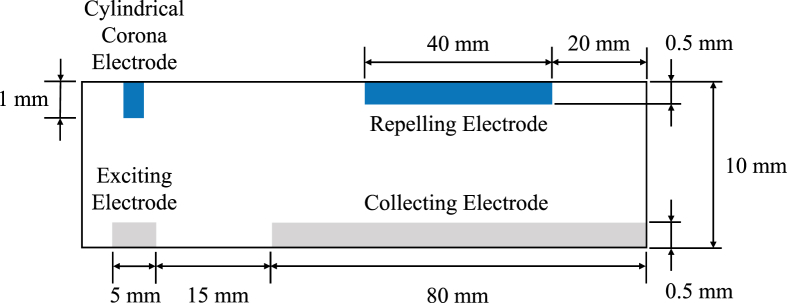
The schematic of the ESP under test (side view). Not drawn to scale.

**Figure 4:**
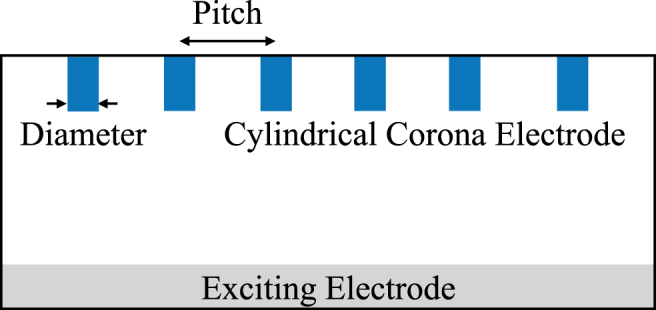
The schematic of the parameters of interest (front view).

The ESP under test is a single channel two-stage ESP, as shown in Figure 3. The schematic and two parameters of the cylindrical corona electrodes this paper concerns, the diameter and the pitch, are shown in Figure 4 and summarized in Table 1, respectively. The enclosure of the ESP under test is 3D printed using PLA. The ESP under test has a width of 140 mm. The cylindrical corona electrodes are evenly distributed across the width of the ESP under test. Therefore, the number of cylindrical corona electrodes changes when the pitch of the cylindrical corona electrodes changes. All the cylindrical corona electrodes are made of high-speed steel and all the plate electrodes (exciting, repelling, and collecting electrodes) are made of aluminum. The reason to make the length of the repelling electrode shorter than that of the collecting electrode is to ensure no corona discharge occurring between the repelling and the collecting electrodes. In the following text, this paper uses the pitch and the diameter to represent the pitch and the diameter of the cylindrical corona electrodes, respectively. Additionally, the cylindrical electrode denotes the cylindrical corona electrode.

**Table 1:** The information of the cylindrical electrode.

Diameter	Pitch (Number of Cylindrical Electrodes)
0.4 mm	10 mm (14), 15 mm (10), 20 mm (7)
0.7 mm	
1.0 mm	

## 3 Results and discussion

### 3.1 Current-voltage characteristic

**Figure 5:**
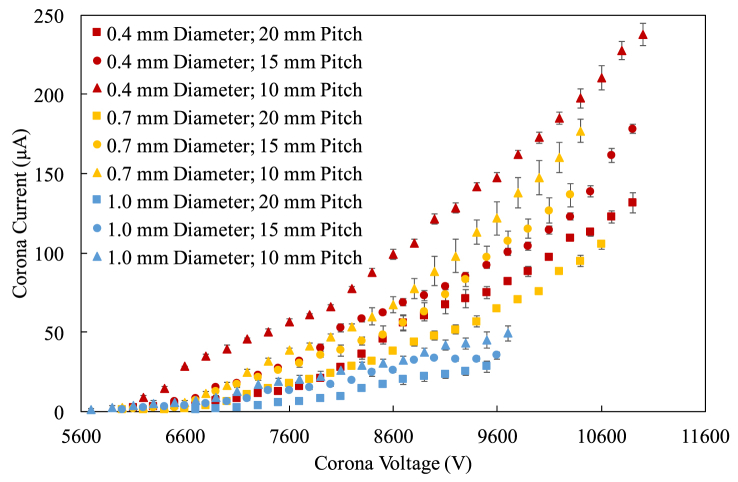
The current-voltage characteristics of the ESPs under test.

Figure 5 shows the characteristic curves of the ESPs under test. Each data point represents an average of three measurements and the error bar is the standard deviation.

Since the current is proportional to the current density [16], the total current drawn by all the corona electrodes should be proportional to the number of the corona electrodes. Therefore, the corona current increases when the pitch decreases, as shown in Figure 5, simply because there are more cylindrical electrodes when the pitch decreases. Besides, thinner cylindrical electrodes have higher sparkover voltages because it was observed that the thinner cylindrical electrodes perform much more stable than the thicker ones do. To be noted that corona discharge happens when the electric field strength falls within the breakdown electric strength (~3.2 × 10^6^ V/m) and a certain high electric strength that makes sparkover, while both strengths are a function of geometrical configurations of the electrodes and the dielectric conditions.

### 3.2 Collection efficiency

**Figure 6:**
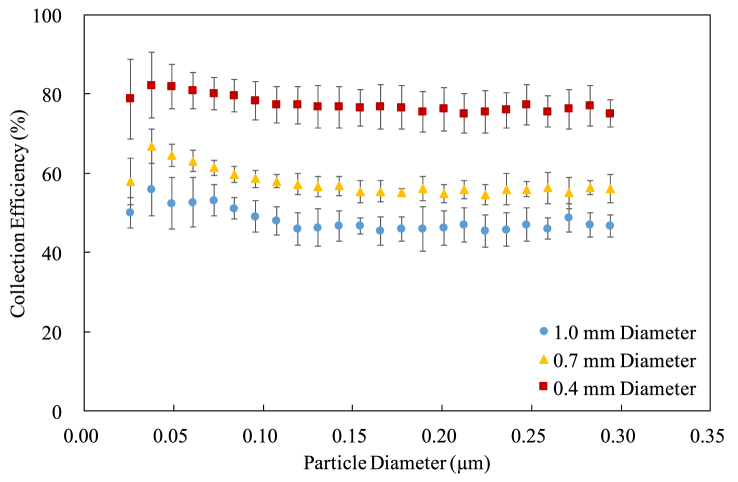
The collection efficiency for the cylindrical electrodes at 10 mm pitch.

**Figure 7:**
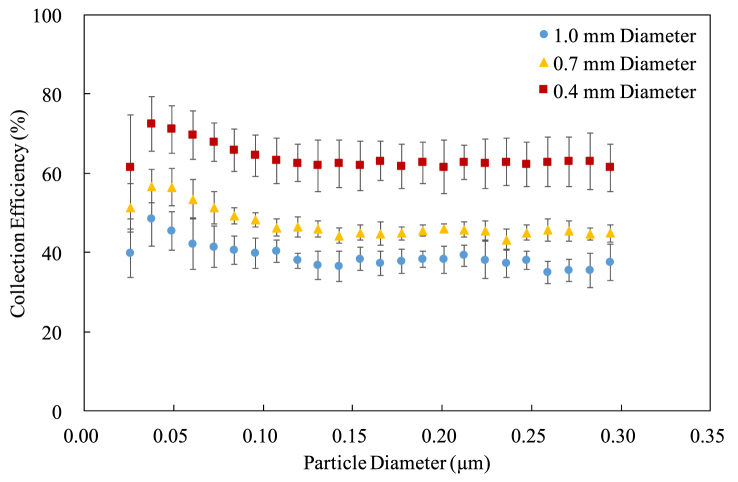
The collection efficiency for the cylindrical electrodes at 15 mm pitch.

**Figure 8:**
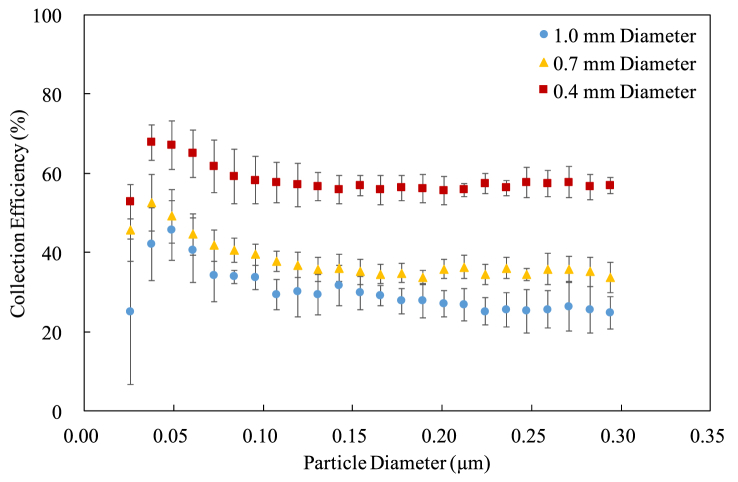
The collection efficiency for the cylindrical electrodes at 20 mm pitch.

Figure 6, Figure 7, and Figure 8 show the collection efficiency in terms of particle size for the pitch of 10 mm, 15 mm, and 20 mm, respectively. Each data point represents an average of seven measurements and the error bar denotes the standard deviation. The corona voltage is 8 kV, while the repelling voltage is 11 kV. Note that these voltages are not optimum for the collection efficiency.

It can be seen that both the diameter and the pitch affect the collection efficiency. Using the thinner cylindrical electrodes results in the higher collection efficiency because the electric fields around a thinner cylindrical electrode are stronger than those around a regular one, making a larger ionization region [11, 13]. In other words, the efficiency of particle charging is high when using thin cylindrical electrodes [17, 18]. Moreover, the collection efficiency seems nonlinearly and inversely proportional to the diameter, suggesting that there exists an optimum diameter to have the best collection efficiency. Regarding the pitch, using the cylindrical electrodes with a shorter pitch also results in higher collection efficiency. This is just because the charger has more cylindrical electrodes when the pitch gets shorter.

The standard deviation is extraordinarily large for the smallest particle because the number of particles is small so that the collection efficiency calculated by (1) varies a lot. That is, a small instrumental deviation leads to a big percentage error. Despite some variations, the standard deviations for other particle sizes are acceptable in a typical engineering standpoint.

### 3.3 Degradation of corona electrode

The degradation testing is conducted by fixing the number of the cylindrical electrodes at four regardless of the pitch, while the collector is disabled. The setup and the operating conditions are exactly the same as stated in the previous sections (8 kV corona voltage, 11 kV repelling voltage, and 4.3×10^−3^ m^3^/s flow rate). Only the cylindrical electrode at the most left-hand side shown in Figure 4 is presented and discussed. There are three stages of the degradation testing. The first stage is to turn the charger on for 24 hours and then take the cylindrical electrodes out of the charger to perform the examinations. The second stage is to reinstate the cylindrical electrodes (got from the first stage) back and keep the charger working for another 48 hours, then perform the same examinations as those done in the first stage. The third stage is to do exactly the same things as those done in the second stage except keeping the charger working for another 72 hours. The examinations include taking SEM photos to visually see how the surface of the cylindrical electrode changes over time and using energy-dispersive X-ray spectroscopy (EDS) to analyze how the surface elements of the cylindrical electrode change. The corona currents are monitored every 10 seconds by a data logger (Fuji-PHR 12B14).

#### 3.3.1 Decreasing of corona current

**Figure 9:**
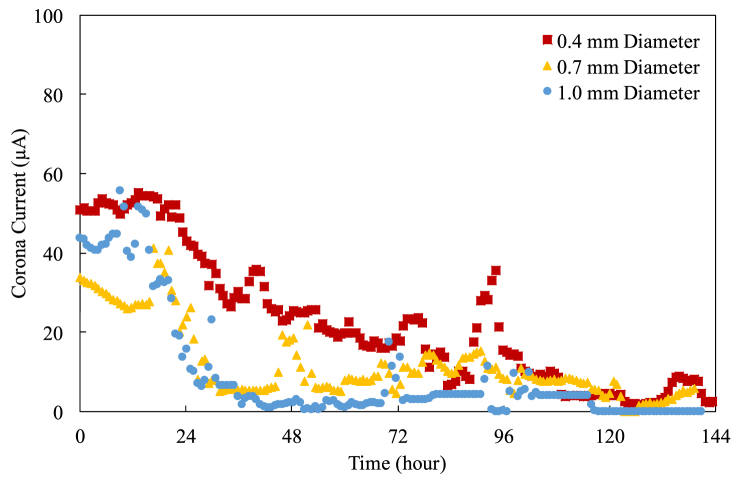
The current-time characteristics for 10 mm pitch.

**Figure 10:**
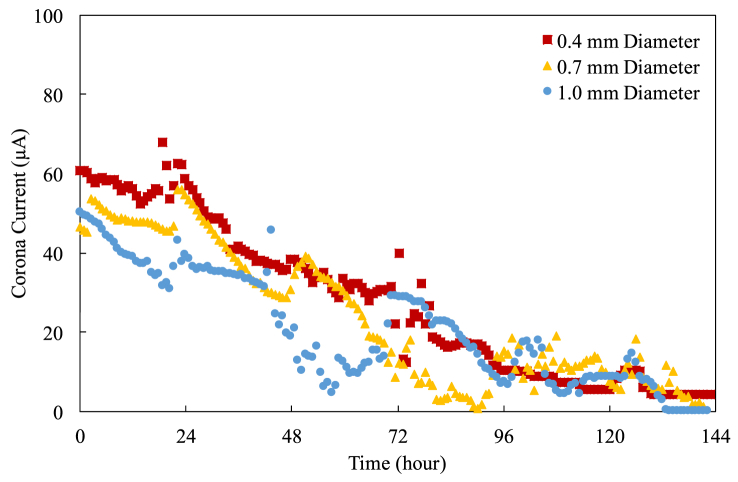
The current-time characteristics for 15 mm pitch.

**Figure 11:**
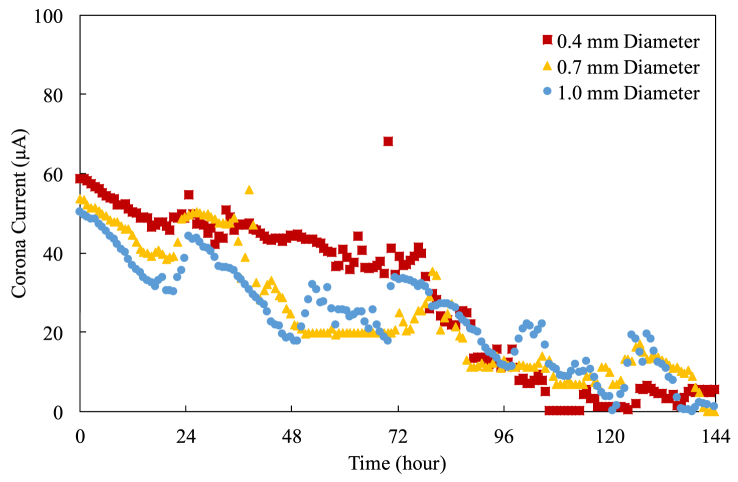
The current-time characteristics for 20 mm pitch.

Figure 9, Figure 10, and Figure 11 show the corona currents versus time for the pitch of 10 mm, 15 mm, and 20 mm, respectively. Each data point is an average of 50-minute measurements.

One can find that the corona current fluctuates significantly and goes to zero eventually. The corona current decreases over time because the cylindrical electrodes and the exciting electrodes oxidize over time, suppressing the electric field (and thus the corona current) because the oxidants have lower dielectric constants [19, 20, 21]. Additionally, there are more and more contaminants settling down on the cylindrical electrode as well, somewhat affecting the surface characteristics of the cylindrical electrode. The fluctuations could attribute to the same reasons mentioned. The pictures of the oxidation and the contaminant deposition are shown in the following section. Please be reminded that this degradation testing is conducted using a fixed number of cylindrical electrodes regardless of the pitch. Thus, the current of the case with a large pitch is higher than that with a small pitch because the electric field of the case with a large pitch is stronger than that with a small pitch [12].

Despite the electric field using a thin cylindrical electrode is stronger at the beginning of the operation, resulting in higher corona current, it is getting difficult to tell which diameter of the cylindrical electrodes performs better when the operation lasts longer. This is because the contaminants deposited on and the oxidants grew on the cylindrical electrodes are enough to impact and to suppress the electric fields considerably. The patterns and the strengths of the electric fields around the cylindrical electrodes might be different from what they were at the beginning of the operation.

The pitch also plays an important role in affecting the corona current. Generally speaking, in the first 24-hour operation, the corona current drains out almost at the same rate regardless of the diameter at the same pitch. Furthermore, the corona current of the case with a shorter pitch keeps effective shorter because the cylindrical electrodes with a shorter pitch have higher discharge energy [13].

#### 3.3.2 SEM photos and EDS results of cylindrical electrode

**Figure 12:**
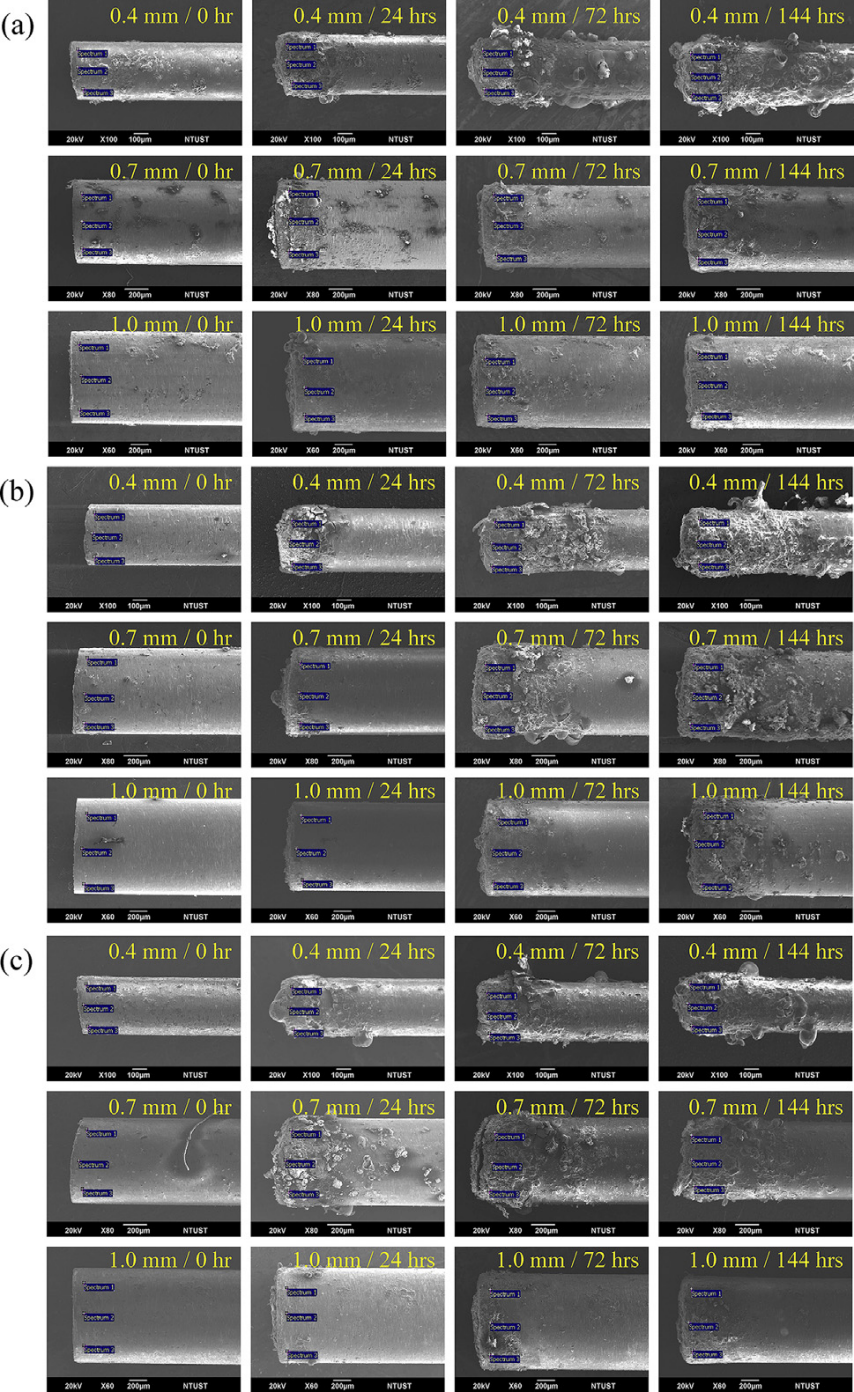
The SEM photos of the tip of the cylindrical electrodes: (a) for 10 mm pitch, (b) for 15 mm pitch, and (c) for 20 mm pitch. The blue stickers are the locations of the EDS analysis shown in Table 2.

To see how the long-term operation affects the surface characteristics of the cylindrical electrode, Figure 12 is the SEM photos and Table 2 shows the element analysis (EDS) that is a three-point average specified in the corresponding SEM photos shown in Figure 12.

**Table 2:** The EDS analysis of the tip of the cylindrical electrodes (round up).

Pitch	Diameter	Element	Hour(s) of Operation			
			0	24	72	144
10 mm	0.4 mm	Fe	51%	59%	56%	49%
		O	10%	30%	44%	49%
		C	30%	4%	0%	0%
	0.7 mm	Fe	55%	62%	53%	41%
		O	4%	22%	47%	54%
		C	30%	12%	0%	0%
	1.0 mm	Fe	54%	74%	55%	54%
		O	5%	16%	39%	41%
		C	31%	3%	0%	0%
15 mm	0.4 mm	Fe	42%	57%	46%	39%
		O	7%	34%	52%	55%
		C	35%	0%	0%	0%
	0.7 mm	Fe	46%	59%	55%	48%
		O	8%	37%	45%	50%
		C	34%	0%	0%	0%
	1.0 mm	Fe	64%	67%	51%	49%
		O	1%	23%	48%	51%
		C	19%	0%	0%	0%
20 mm	0.4 mm	Fe	25%	51%	50%	40%
		O	12%	39%	47%	57%
		C	35%	7%	0%	0%
	0.7 mm	Fe	60%	65%	60%	46%
		O	6%	30%	40%	51%
		C	23%	0%	0%	0%
	1.0 mm	Fe	55%	74%	60%	50%
		O	5%	18%	39%	48%
		C	32%	0%	0%	0%

The SEM photos show that the surface of the cylindrical electrode deteriorates over time. There are many unknown contaminants (possibly aerosol debris) deposit on the cylindrical electrode in addition to the oxidants. When the cylindrical electrode is thinner, the electric field is stronger, and thus, there are more the oxidants that can deteriorate the cylindrical electrode. Although it is difficult to evaluate the effects on the electrode degradation contributed to the pitch by just looking at the SEM photos, it has been shown that the ionization region around a single cylindrical electrode is proportional to the pitch of the cylindrical electrodes [13].

The EDS results reveal that the content of oxygen keeps increasing over time, ramping up to ~50% among all these cases. The content of carbon decreases down to zero rapidly after ~24 h operation because the oxidations usually accompany the decarburization [22]. At the same pitch, the content of oxygen also increases when the diameter gets smaller because the electric field gets stronger. Yet, it still presents some difficulties in identifying the trends caused by the pitch. The reason could be that the pitches selected are not diverse enough to create a significant variation.

## 4 Conclusions

This paper presents the characteristics of the ESPs that use cylindrical corona electrodes whose longitudinal axis is perpendicular to the exciting electrodes. The current-voltage curve and the collection efficiency with respect to the pitch and the diameter of the cylindrical corona electrodes are demonstrated. This paper also discusses how the surface characteristics of the cylindrical corona electrodes change over time and how such changes degrade the corona current of the cylindrical corona electrodes.

The results show that the electric field around the cylindrical corona electrodes dominates the characteristics presented. When the cylindrical corona electrode is thinner, the electric field around the cylindrical corona electrode is stronger. When the electric field around the cylindrical corona electrode is stronger, the collection efficiency is higher, and the oxidation on the cylindrical corona electrodes is severer. Despite the pitches discussed have no clear impacts on the oxidation, the performance of the cylindrical corona electrodes with a shorter pitch deteriorates faster because of the stronger corona discharge energy. The results also imply that there is a trade-off between the pitch and the diameter of the cylindrical corona electrodes for getting the best collection efficiency and the longest life of the cylindrical corona electrodes.

## Declarations

### Author contribution statement

Tsrong-Yi Wen: Conceived and designed the experiments; Analyzed and interpreted the data; Contributed reagents, materials, analysis tools or data; Wrote the paper.

Jiann-Lin Su: Conceived and designed the experiments; Performed the experiments; Analyzed and interpreted the data; Contributed reagents, materials, analysis tools or data.

### Funding statement

This work was financially supported by the High Speed 3D Printing Research Center from the Featured Areas Research Center Program within the framework of the Higher Education Sprout Project by the Ministry of Education (MOE) in Taiwan. This work was also supported by the Ministry of Science and Technology, Taiwan (MOST 105-2218-E-011-012).

### Competing interest statement

The authors declare no conflict of interest.

### Additional information

No additional information is available for this paper.
